# Enhancing stock volatility prediction with the AO-GARCH-MIDAS model

**DOI:** 10.1371/journal.pone.0305420

**Published:** 2024-06-11

**Authors:** Ting Liu, Weichong Choo, Matemilola Bolaji Tunde, Cheongkin Wan, Yifan Liang

**Affiliations:** 1 School of Business and Economics, Universiti Putra Malaysia, Seri Kembangan, Malaysia; 2 Faculty of Accountancy, Department of Economics and Corporate Administration, Finance and Business, Setapak, Malaysia; Loughborough University, UNITED KINGDOM

## Abstract

Research has substantiated that the presence of outliers in data usually introduces additional errors and biases, which typically leads to a degradation in the precision of volatility forecasts. However, correcting outliers can mitigate these adverse effects. This study corrects the additive outliers through a weighting method and let these corrected values to replace the original outliers. Then, the model parameters are re-estimated based on this new return series. This approach reduces the extent to which outliers distort volatility estimates, allowing the model to better adapt to market conditions and improving the accuracy of volatility forecasts. This study introduces this approach for the first time to generalized autoregressive conditional heteroskedasticity mixed data sampling (GARCH-MIDAS) models, so as to establish an additional outliers corrected GARCH-MIDAS model (AO-GARCH-MIDAS). This pioneering approach marks a unique innovation. The research employs a diverse array of evaluation methods to validate the model’s robustness and consistently demonstrates its dependable performance. Findings unequivocally reveal the substantial influence of outliers on the model’s predictive capacity, with the AO-GARCH-MIDAS model exhibiting consistent superiority across all evaluation criteria. Additionally, while the GARCH model showcases stronger estimation capabilities compared to the GARCH-MIDAS model, the latter demonstrates heightened predictive prowess. Notably, regarding variable selection, the results underscore the greater predictive informational value inherent in realized volatility over other low-frequency factors.

## 1. Introduction

Amidst the deepening facets of economic globalization and liberalization, the prominence of financial markets has notably heightened within the contemporary market economy, particularly evident in the ascendancy of stock markets. This trend manifests through the increasing prevalence of index trading within stock markets, positioning these markets as pivotal and highly coveted investment avenues within the global financial domain [1]. Concurrently, volatility, serving as a fundamental metric of equity risk, assumes a pivotal role not solely within the investment sphere but also exerts substantial influence on asset valuation, risk mitigation strategies, and the formulation of macroeconomic policies [2]. The adept comprehension and precise prognostication of stock market volatility hold multifaceted significance and practical implications. Notably, such endeavors can aid investors in devising astute investment frameworks, furnish market participants with mechanisms to preclude market-related risks, and equip policymakers with crucial benchmarks to ensure the smooth operation of the national economy [3]. Especially for investors, reducing errors in forecasting stocks can reduce investment risk and increase profitability [4].

In the realm of prediction, the presence of outliers cannot be disregarded if heightened prediction accuracy is sought. Outliers, denoting data points significantly deviating from the general data sample [5]. Notably, these outlier observations may introduce inaccuracies in both model parameter estimation and volatility prediction [6]. This occurrence emanates from the model’s tendency to overly emphasize this anomalous information during estimation, consequently resulting in forecasting overfitting and subsequently generating less reliable volatility forecasts. Because of their inevitability, particularly within financial contexts such as time series data of stock markets and asset prices, addressing the adverse effects arising from outliers becomes imperative.

To tackle this challenge, researchers have embraced diverse methodologies in addressing financial time series volatility forecasting. Franses and Ghijsels [7] introduce the additive outlier-corrected returns method within the Generalized Autoregressive Conditional Heteroskedastic (GARCH) model. Specifically, the time series model is first fitted, and the residual series of the model is calculated (the residual series being the difference between the observed values and the fitted values of the model). Then, the residual series is standardized, meaning the standardized residuals of the residuals are calculated. Statistical methods are utilized to detect outliers in the standardized residuals, identifying values that fall outside certain boundaries by setting thresholds. Once the outliers are detected, they are corrected using a weighting method that adjusts them according to their nature and magnitude of impact, thus reducing their influence on the volatility estimate. After all the additive outliers in the model have been corrected, the model parameters are re-estimated based on this new return series. This approach diminishes the extent to which outliers distort volatility estimates, enhancing the stability of volatility estimates and, consequently, improving the robustness of the model during forecasting, thereby rendering the forecasts more reliable. Affirming its efficacy across four distinct stock markets in enhancing stock volatility.

This study collectively underscore that the presence of outliers introduces bias in volatility forecasting, necessitating their identification and rectification as pivotal. This study aims to profoundly explore strategies for handling outliers, referencing the work of Franses and Ghijsels [7] extensively. Notably, this methodology is grounded in the GARCH model. Nonetheless, this model’s limitation in capturing volatility solely on a single time scale is evident [8]. To overcome this limitation, the GARCH-MIDAS (Mixed Data Sampling) model, an extension within the GARCH framework pioneered by Engle, Ghysels [9], compensates for this shortfall. This model not only accommodates independent and dependent variables of different frequencies but also retains the fidelity of high-frequency data without altering its native frequency, preserving its realism and effectiveness. Additionally, the GARCH-MIDAS model comprehensively captures both short-term and long-term components of aggregate stock market volatility. However, akin to the GARCH model, it grapples with data featuring excessive kurtosis. In essence, this limitation can result in models compromising their fitting accuracy to sufficiently accommodate current values when encountering outliers. This is particularly evident when dealing with outliers such as additive outliers, which exert influence not only on the individual observations they pertain to but also on surrounding data points [10,11]. In such scenarios, the efficacy of GARCH-MIDAS is constrained. Building upon the GARCH-MIDAS model, this paper introduces a corrected outlier function inspired by the work of Franses and Ghijsels [7], culminating in the additive outlier GARCH-MIDAS (AO-GARCH-MIDAS) model. This innovative framework heralds a new era in volatility forecasting, aiming to rectify distortions induced by outliers and augment forecasting precision.

Since the GARCH-MIDAS model is used to model the relationship between data of different frequencies, only high-frequency stock data is not sufficient. Therefore, low-frequency data also need to be introduced into the model to understand the mechanism of stock market volatility more comprehensively. Without considering other influences, realized volatility (RV) is usually chosen as the low-frequency data. RV is a measure of asset price movements and represents the actual volatility over time. In addition to RV, a range of macroeconomic variables can be selected as low-frequency data. These macroeconomic variables reflect changes in the economic environment and may have a significant impact on stock market volatility.

As an intricate derivative component of the real economy, stock market volatility manifests as a complex interplay of diverse determinants. A multitude of empirical inquiries have yielded substantive insights into the potential causative factors linked to macroeconomic variables within the ambit of the stock market. Noteworthy contributions by Asgharian, Hou [12] and Song, Tang [13] affirm the efficacy of macroeconomic variables in explicating stock market volatility. Their research also underscores that the low-frequency macroeconomic data augments the prognostic potency of the model. The macroeconomic metric, money supply (M2), being considered pivotal in impacting national income and stock prices [14]. In an empirical analysis, Ma, Yang [15] assert that the M2 exhibits a significant and positive correlation with stock market volatility in China, utilizing the GARCH-MIDAS model. Furthermore, Bhuiyan and Chowdhury [16] employ Vector Error Correction and artificial neural networks models to demonstrate the long-term cointegration of money supply with the US stock market, along with a positive correlation. Notably, M2 exhibits robust predictive capabilities, as underscored in the forecasting [17]. Similarly, the impact of exchange rates (ER) on stock markets has piqued the interest of researchers. Aslam [18] confirms a causal relationship between ER and stocks. Studies by Sensoy and Sobaci [19] and Endri, Abidin [20] further establish a positive relationship between exchange rate and the stock market. However, some research indicates that ER can exert a significant negative impact on stock returns [21,22]. Dai, Zhou [23] assert the formidable predictive power of exchange rates within the framework of forecasting. These findings collectively illuminate the intricate relationship between macroeconomic indicators, particularly M2 and exchange rates, and stock market dynamics, constituting a noteworthy area of scholarly investigation. Furthermore, economic policy uncertainty (EPU) has garnered considerable scholarly attention in recent years. it is regarded as an influential indicator explaining stock volatility [24]. The results of a large number of scholars support the conclusion that EPU has a significant effect on stock volatility [25–27]. Therefore, these three variables are chosen as predictors of stock volatility in the present study. EPU also shows good predictive ability in forecasting [26,28–30]. In alignment with the framework presented herein, this study discerningly selects M2, ER, and EPU as pivotal drivers underpinning the examination of stock market volatility dynamics.

This study has opted to focus on the stock markets of China and Japan. The rationale behind this choice is twofold. Firstly, these markets serve as pertinent reflections of the developmental trajectory and distinctive attributes inherent within the Asian economic landscape. Secondly, the stock markets of China and Japan command significant stature not only within the confines of the Asian region but also globally, thus warranting meticulous scholarly attention.

This study’s primary contribution lies in the novel application of a multivariate additive outlier GARCH-MIDAS model, significantly enhancing the accuracy of stock volatility forecasting. The findings demonstrate robustness across diverse evaluation criteria, affirming the model’s efficacy. On one hand, the novel model addresses the constraint that the correction for additive outliers is restricted to GARCH models, by extending the methodology of Franses and Ghijsels [7] to encompass GARCH-MIDAS models. On the other hand, it enhances the robustness of the GARCH-MIDAS model introduced by Engle, Ghysels [9], enabling it to more effectively manage the complex scenario of additive outliers. Additionally, based on the MCS results, the result shows that the introduction of macroeconomic indicators into the GARCH-MIDAS model can effectively improve the prediction of stock market volatility. Specifically, the forecasting performance using the GARCH-MIDAS-RV-X model is significantly better than the GARCH-MIDAS-RV model alone. This finding further supports [12] that the inclusion of low-frequency macroeconomic information in the GARCH-MIDAS model improves the model’s forecasting accuracy. Song, Tang [13] also argue that both before and after the introduction of macroeconomic variables, the model’s forecasting ability is significantly improved. Notably, this study points out that the same economic indicator can have different impacts on the stock markets of different countries. Humpe and Macmillan [31] also find this phenomenon when they investigate the performance of the U.S. and Japanese stock markets on the same indicator, and they explain that this variability could be attributed to the structure of the economies of the two countries, the policy environments, and the behaviors of the market participants.

Following the introductory chapter, the remaining chapters of this study are organized as follows. Chapter 2 details the construction process of AO-GARCH-MIDAS. Chapter 3 presents the descriptive statistics of the data and the results of the correlation tests. Chapter 4 describes the in-sample and out-of-sample experimental results. Finally, Chapter 5 concludes the study.

## 2. Methodology

The pivotal innovation within this study hinges upon the pioneering work of Franses and Ghijsels [7], which extends the application of additive outliers to the GARCH-MIDAS framework. The GARCH-MIDAS model utilized in this study draws its foundation from the work of Engle, Ghysels [9] and Engle and Rangel [32]. Assuming *r*_*i*,*t*_ is the logarithmic rate of return on day i of month t, the characterization of volatility within the framework of the GARCH-MIDAS model can be articulated as follows

$$r_{i,t}-E_{i-1,t}(r_{i,t})=\sqrt{\tau_{t}g_{i,t}}\varepsilon_{i,t},E_{i-1,t}(r_{i,t})=\mu ,\forall i=1,2,\cdots ,N_{t}$$ Eq 1

$$\varepsilon_{i,t}|\psi_{i-1,t}∼N(0,1),\;\sigma_{i,t}^{2}=\tau_{i}g_{i,t}$$ Eq 2

In Eq 1, the expression for volatility is decomposed into two components: the short-term volatility *g*_*i*,*t*_, which satisfies to the GARCH (1, 1) model, and long-term volatility *τ*_*i*_. *E*_*i*−1,*t*_ denotes the conditional expectation while *ε*_*i*,*t*_ represents the random disturbance term, assumed to follow a standard normal distribution. *N*_*t*_ signifies the number of days in month t. *ψ*_*i*−1,*t*_ in Eq 2 delineates the information set pertaining to the i-1 day of the rate of return in month t.

The long-term component *τ*_*i*_ is delineated through the incorporation of diverse low-frequency variables, encompassing factors such as realized volatility (RV), M2, ER, and EPU. To investigate the impact of these factors on long-term component, this study explores three specifications. The first specification of Eq 3 exclusively incorporates RV, denoted as GARCH-MIDAS-RV. The second specification includes three variables: M2, ER, and EPU, designated as GARCH-MIDAS-X (see Eq 4). The third specification incorporates both RV and all three macroeconomic variables, denoted as GARCH-MIDAS-RV-X (see Eq 5). These specifications are formally articulated as follows:

$$\tau_{t}=m+\theta_{RV}\sum_{k=1}^{k}\varphi_{k}(\omega_{1},\omega_{2})RV_{t-k}$$ Eq 3

$$\tau_{t}=m+\theta_{M2}\sum_{k=1}^{k}\varphi_{k}(\omega_{1},\omega_{2}){M2}_{t-k}+\theta_{ER}\sum_{k=1}^{k}\varphi_{k}(\omega_{1},\omega_{2}){ER}_{t-k}+\theta_{EPU}\sum_{k=1}^{k}\varphi_{k}(\omega_{1},\omega_{2}){EPU}_{t-k}$$ Eq 4

$$\tau_{t}=m+\theta_{RV}\sum_{k=1}^{k}\varphi_{k}(\omega_{1},\omega_{2})RV_{t-k}+\theta_{M2}\sum_{k=1}^{k}\varphi_{k}(\omega_{1},\omega_{2}){M2}_{t-k}+\theta_{ER}\sum_{k=1}^{k}\varphi_{k}(\omega_{1},\omega_{2}){ER}_{t-k}+\theta_{EPU}\sum_{k=1}^{k}\varphi_{k}(\omega_{1},\omega_{2}){EPU}_{t-k}$$ Eq 5

Where *RV*_*t*_ represents the fixed time span RV at time t, which can be written as $RV_{t}=\sum_{i=1}^{N_{t}}r_{i,t}^{2}$. In addition, k denotes the maximum lag order of low-frequency variables, selected by AIC and BIC information standards. *φ*_*k*_(*ω*_1_,*ω*_2_) is the weight scheme of the Beta lag structure [9], because it is more flexible and more commonly used to accommodate various lag structures [33], the polynomial shows as below

$$\varphi_{k}(\omega_{1},\omega_{2})=\frac{(\kappa /K{)}^{\omega_{1}-1}(1-(\kappa /K{)}^{\omega_{2}-1}}{\sum_{j=1}^{K}(j/K{)}^{\omega_{1}-1}(1-j/K{)}^{\omega_{2}-1}}$$ Eq 6

Fix *ω*_1_ = 1, in order to ensure that the weight of the lag variable is in the form of attenuation. In other words, the closer the distance to the current period, the greater the impact on the current period (Yaya et al., 2022). The coefficient determines the attenuation speed of the impact of low-frequency data on high-frequency data. Therefore, the polynomial can be simplified as

$$\varphi_{k}(\omega_{2})=\frac{(1-\kappa /K{)}^{\omega_{2}-1}}{\sum_{j=1}^{K}(1-j/k{)}^{\omega_{2}-1}}$$ Eq 7

The focus of this section is to combine AOs with GARCH-MIDAS model. The enhancement of the conventional GARCH-MIDAS model through the rectification of Aos aims to refine the model’s predictive capacity and accuracy within the realm of financial econometrics, the equation can be rewritten as

$$\frac{r_{i,t}^{2}}{\tau_{t}}=g_{i,t}+z_{i,t}$$ Eq 8

$$E_{t-1}(z_{i,t})=0$$ Eq 9

Based on the above formula rewrite the Short-term volatility *g*_*i*,*t*_ as

$$\frac{r_{i,t}^{2}}{\tau_{t}}-z_{i,t}=(1-\alpha -\beta )+\alpha \frac{(r_{i-1,t}-\mu {)}^{2}}{\tau_{t}}+\beta (\frac{r_{i,t}^{2}}{\tau_{t}}-z_{i-1,t})$$ Eq 10

This formula corresponds to the paper of Franses and Ghijsels [7] on GARCH (1, 1) model for $r_{i,t}^{2}$.


$$Let\;f_{i,t}=\frac{r_{i,t}^{2}}{\tau_{t}}$$

$$(1-(\alpha +\beta )L{)f}_{i,t}=(1-\beta L)z_{i,t}$$ Eq 11

From this equation, *ϕ*(*L*) and *θ*(*L*) can be determined as below

ϕ(L)=1−(α+β)L Eq 12

θ(L)=1−βL Eq 13

According to the equation ${r_{t}^{*}}^{2}={\hat{z}}_{t}^{*}+{\hat{h}}_{t}$ form Franses and Ghijsels [7], the formula of ${r_{i,t}^{*}}^{2}$ can be constructed as follow

$$r_{i,t}^{*2}=\tau_{t}(z_{i,t}^{*}+g_{i,t})\;t=\nu$$ Eq 14

Hence, the AO-corrected returns can be constructed

$$r_{i,t}^{*}=r_{i,t}\;t\neq \nu$$ Eq 15

$$r_{i,t}^{*}=sign(r_{i,t}).{\left({r_{i,t}^{*}}^{2}\right)}^{1/2}\;t=\nu$$ Eq 16

This expression shows that although *r*_*i*,*t*_ is replaced, its sign is retained in $r_{i,t}^{*}$, when *t* = *ν*.

Based on Chen and Liu [34] of AO-ARMA, the estimated residuals ${\hat{\varepsilon }}_{t}$ can be represented by

$${\hat{\varepsilon }}_{t}=\pi (L)y_{t}$$ Eq 17

When

$$t<\nu ,\;x_{t}=0$$

$$t=\nu ,\;x_{t}=1$$

$$t=\nu +i(i>0),\;x_{t+i}=-\pi_{i}$$

At time *t* = *ν*, the impact *ρ* of AO can be estimated as

$$\hat{\rho }(\nu )=\frac{\sum_{t=\nu }^{n}x_{t}{\hat{\varepsilon }}_{t}}{\sum_{t=\nu }^{n}x_{t}^{2}}$$ Eq 18

To test the significance of AO model, Chang, Tiao [35] propose to standardize $\hat{\rho }(\nu )$. It requires an estimate of the variance of the residual process, this estimate should ideally not contain too much bias because of outliers. This study uses the method of Chen and Liu [34] the so-called ‘omit one’ to estimate a robust error variance. Based on this approach, we can get a standardized statistic

$$\hat{\nu }=\frac{\hat{\rho }(\nu )}{{\hat{\sigma }}_{a}}\sqrt{\sum_{t=\nu }^{n}x_{t}^{2}}$$ Eq 19

The influence of AO is significant when $\hat{\nu }$ exceeds the value C. As Franses and Ghijsels [7] mentioned, $\hat{\nu }$ is asymptotically standard normal. As posited by Chen and Liu [34], it is imperative to scrutinize the parameter C for values exceeding 3 when the dataset comprises more than 200 observations. Although other choices for C are viable, this study has identified superior outcomes when C equals 4. When the value of $\hat{\nu }$ exceeds the value C, and *t* = *ν*, the observation *y*_*t*_ shall be substituted with AO-corrected $y_{t}^{*}$, derived from Eq 18, and the additive outlier model $y_{t}=y_{t}^{*}+\rho I_{t}(\nu )$.

In the dataset, to avoid the existence of multiple AOs, these steps need to repeat unless $\hat{\nu }$ becomes insignificant. When there is no more additive outlier, the final step is to re-estimate the model parameters based on all observations, where some of them have been corrected by using AO model.

## 3. Data description and preliminary analysis

**Fig 1:**
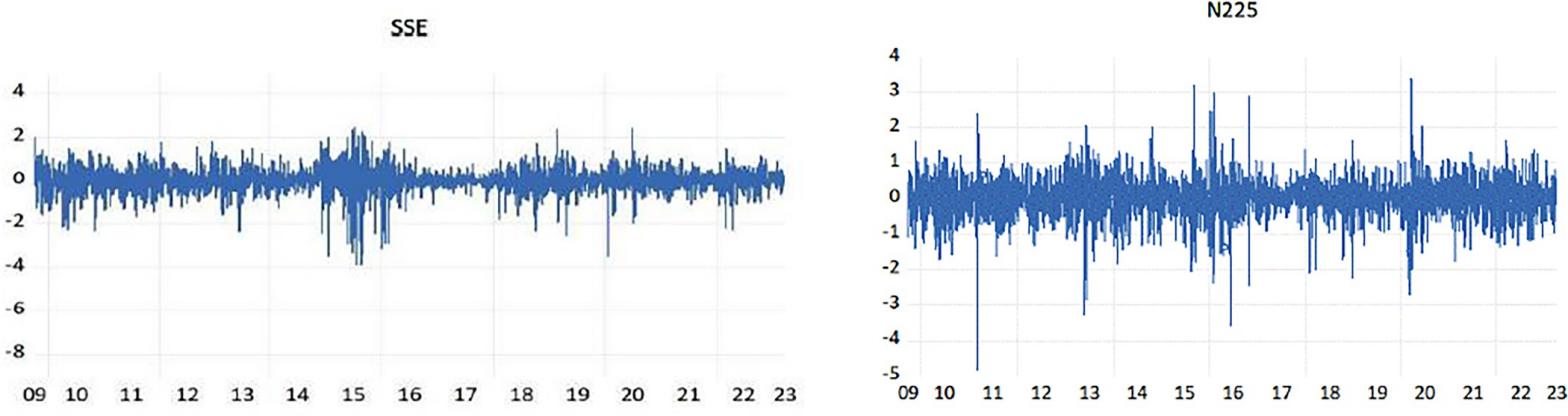
SSE和N225 stock return series.

Our study is centered on the examination of the Chinese and Japanese stock markets, where we have gathered data from daily closing prices of the Shanghai Stock Exchange Composite Index, comprising 3,278 observations, and the Nikkei 225 with 3,298 observations. This data spans from October 1, 2009, to March 31, 2023, and has been sourced from Yahoo Finance (https://finance.yahoo.com). The log returns for the closing prices of these two stocks are computed and labeled as SSE and N225, respectively. Additionally, monthly data encompassing M2, exchange rates (ER), and Economic Policy Uncertainty (EPU) variables span from October 2009 to March 2023, each variable comprising 162 observations. Chinese money supply data is obtained from the People’s Bank of China, while Japanese M2 data is sourced from the Bank of Japan. Exchange rates (ER) data of China and Japan are retrieved from the Federal Reserve Economic Data (FRED, https://fred.stlouisfed.org). The exchange rates are calculated using the Renminbi (RMB) against the US dollar and the Japanese yen (JPY) against the US dollar, respectively. It is noteworthy that the frequency employed for this indicator in this study is monthly, primarily due to its application within the GARCH-MIDAS model, and MIDAS polynomial, which is applied to macroeconomic or financial variables at monthly, quarterly, or biannual frequencies [36]. Economic Policy Uncertainty (EPU) is constructed through a systematic approach, involving the classification and tallying of articles containing keywords such as economics, policy, and uncertainty. This is complemented by the application of a series of standardized steps, following the methodology outlined by [37]. The EPU series is sourced from the Economic Policy Uncertainty Website (http://www.policyuncertainty.com). For ease of identification, variables originating from China and Japan are distinctly marked with prefixes ’C’ and ’J’ respectively. These return series are visually represented in Fig 1. Upon observation, overall stability characterizes each series, albeit with occasional notable deviations.

Table 1 is instrumental in furnishing a holistic overview, encompassing descriptive statistics, stability examinations, and heterogeneity assessments for both Chinese and Japanese stock returns, in conjunction with each pertinent economic variable. In this study, the rate of change of M2, ER and EPU is expressed as the log difference of the variable. The findings derived from the scrutiny of Skewness, Kurtosis, and the Jarque-Bera (JB) test illuminate salient features characterizing the probability distributions governing SSE, N225, and JEPU. These probability distributions are notably distinguished by the presence of pronounced peaks and pronounced tails skewed toward the left. In contrast, the probability distributions of CER, JM2, and JER exhibit a distinct profile characterized by a rightward shift, spiked configurations, and thick trailing tails. In addition, none of these six datasets conform to a normal distribution. However, CM2 and CEPU exhibit distributions that lack overt features violating the assumptions of normality. The examination of stability through the Augmented Dickey-Fuller (ADF) test, Phillips-Perron (PP) test, and Kwiatkowski-Phillips-Schmidt-Shin (KPSS) test, specifically within the context of unit root detection, yields statistically significant results for all datasets, except for CM2, signifying the robust stability exhibited by these respective data sequences. Notably, the first-order differenced series of CM2 (DCM2) successfully passes the test of stability. Furthermore, an auxiliary analysis is conducted through the application of Engle’s ARCH test to the regression series of SSE and N225 stocks. The outcome reveals a 1% rejection rate of the null hypothesis, suggesting the existence of pronounced heterogeneous effects within these datasets. This outcome underscores the appropriateness of employing a GARCH-type model to effectively capture and model the volatility inherent in the Chinese and Japanese stock markets across these two distinct stock exchanges. The Ljung-Box Q-statistics are also presented in the table, and this autocorrelation test shows significant p-values for SSE, indicating the presence of autocorrelation in the residual series. Specifically, the volatility of its time period receives a significant influence from the prior period volatility. Hence the problem can be correctly handled using GARCH model.

**Table 1:** Descriptive statistics and stationary testing and heteroskedastic test.

Panel A Descriptive statistics							
	Mean	Min	Max	Std. Dev.	Skewness	Kurtosis	P(JB)
SSE	0.002165	-3.853454	2.433618	0.568242	-0.883691	9.079544	0.000000
CM2	0.420960	-0.554221	1.593245	0.416307	0.323569	2.987790	0.243190
CER	0.002472	-1.285742	1.772869	0.436466	0.847789	6.166941	0.000000
CEPU	0.267899	-41.95545	36.99400	14.5854	-0.117788	2.884273	0.792549
N225	0.013404	-4.843875	3.393352	0.571788	-0.391549	7.872514	0.000000
JM2	0.126239	-0.261412	0.889751	0.200436	1.186965	4.899548	0.000000
JER	0.102262	-2.358774	3.204788	0.931771	0.614664	3.993229	0.000218
JEPU	0.010640	-26.96262	25.18561	8.110779	-0.110295	4.055536	0.019752
	Panel B Stationary and heteroskedastic test						
	ADF	PP	KPSS			Ljung-Box Q-statistic (36)	ARCH
SSE	-55.68574*** (0.0001)	-55.69187*** (0.0001)	0.046999			219.9500*** (0.0000)	129.0873*** (0.0000)
CM2	-2.426965 (0.1361)	-15.19483*** (0.0000)	0.758599***				
DCM2	-11.58947*** (0.0000)	-109.9935 (0.0001)	0.196415				
CER	-7.502075*** (0.0000)	-7.207522*** (0.0000)	0.133526				
CEPU	-11.83482*** (0.0000)	-33.61563*** (0.0001)	0.138266				
N225	-58.76959*** (0.0001)	-58.82644*** (0.0001)	0.043331			29.7300 (0.7600)	290.6381*** (0.0000)
JM2	-2.793088* (0.0617)	-9.933491*** (0.0000)	0.133201				
JER	-9.030035*** (0.0000)	-8.997714*** (0.0000)	0.096680				
JEPU	-14.36969 (0.0000)	-28.94255*** (0.0001)	0.209168				

Notes: The values in panel B are the t-statistics for stationary test.

*Indicate rejections of the null hypothesis at the 10% significance level.

**Indicate rejections of the null hypothesis at the 5% significance level.

***Indicate rejections of the null hypothesis at the 1% significance level. The numbers in parentheses are the p-values of the tests.

## 4. Empirical results

### 4.1 In-sample estimation of GARCH-type models

The in-sample estimation results are presented comprehensively in Table 2. Firstly, the parameter *μ* denotes the unconditional mean of stock returns. Secondly, both estimated coefficients *α* and *β*, associated with the total daily volatility of short-term component (*g*_*i*,*t*_) stock returns, exhibit significant positive values at the 1% level across all models. These parameters correspond to short-term components linked to ARCH and GARCH terms, respectively. Their cumulative sum, *α* plus *β*, closely approximates 1, indicating a pronounced volatility persistence effect for both SEE and N225. Further parameters, *ω*_1_ and *ω*_2_, serve as the *β* polynomial weights for the long-run components within the model. Their significance across most variables underscores the predictive capacity of macroeconomic variables and the Economic Policy Uncertainty (EPU) in determining long-run volatility. Additionally, *θ* represents the aggregated weighted rolling window realized volatility for each variable, showcasing varied performance across countries and models.

**Table 2:** Estimated parameters of the GARCH-type model in the Chinese and Japanese samples.

	GARCH	GARCH-MIDAS-RV	GARCH-MIDAS-X	GARCH-MIDAS-RV-X	AO-GARCH-MIDAS-RV-X
*α*	0.0676***(0.0153)	0.0771***0.0190)	0.0609***(0.0156)	0.0737***(0.0171)	0.0310***(0.0067)
*β*	0.9289***(0.0162)	0.9146***(0.0207)	0.9302***(0.0186)	0.8965***(0.0285)	0.9620***(0.0085)
*μ*	0.0049(0.0077)	0.0115(0.0082)	0.0123(0.0083)	0.0113(0.0080)	0.0207***(0.0064)
*m*	-0.4627(0.5893)	-1.0621**(0.3523)	-1.3474***(0.3002)	-2.4890***(0.4189)	-2.4935***(0.1616)
*θ*_*CRV*_		3.3037e-07(0.0009)		0.5078***(0.1565)	0.1737***(0.0390)
*θ*_*CM*2_			0.4850(0.3626)	0.2522**(0.1134)	0.4407**(0.1988)
*θ*_*CER*_			-1.7727**(0.7613)	-0.4986*(0.3019)	-1.7800***(0.6481)
*θ*_*CEPU*_			-0.3246*(0.1799)	-0.1637**(0.0745)	-0.1626**(0.0741)
$\omega_{2}^{CRV}$		4.7626(29.3466)		2.3264***(0.7222)	40.8121(32.9097)
$\omega_{2}^{CM2}$			8.6611**(4.3215)	114.1349***(33.7489)	4.4400***(1.3803)
$\omega_{2}^{CER}$			1.0000*(0.6003)	2.0733**(0.9721)	1.4747***(0.3720)
$\omega_{2}^{CEPU}$			1.0000***(0.2094)	1.0875***(0.3222)	1.0000***(0.2075)
*α*	0.1259***(0.0219)	0.1266***(0.0217)	0.1197***(0.0257)	0.1160***(0.0206)	0.0783***(0.0166)
*β*	0.8295***(0.0298)	0.8033***(0.0397)	0.8276***(0.0439)	0.8013***(0.0416)	0.8410***(0.0416)
*μ*	0.02762***(0.0086)	0.0312***(0.0096)	0.0312***(0.0095)	0.0294***(0.0088)	0.0364***(0.0074)
*m*	-1.0662***(0.1555)	-1.9139***(0.3741)	-1.0078***(0.2299)	-1.9788***(0.3984)	-3.0070***(0.2551)
*θ*_*JRV*_		0.3197**(0.1405)		0.3203*0.1675	0.3364***(0.0962)
*θ*_*JM*2_			-1.3528(1.2378)	-0.3949(0.6591)	2.2797*(1.2840)
*θ*_*JER*_			0.3482*(0.2024)	0.3206**(0.1424)	0.6033**(0.2203)
*θ*_*JEPU*_			0.0741*(0.0422)	0.0649*(0.0334)	0.0531**(0.0241)
$\omega_{2}^{JRV}$		14.0204**(6.3792)		2.8201(2.0210)	12.8771**(4.8761)
$\omega_{2}^{JM2}$			1.0001(0.6506)	1.2480(1.2739)	1.0007***(0.2520)
$\omega_{2}^{JER}$			8.9769(5.5246)	8.6838**(4.0019)	9.2348(7.0097)
$\omega_{2}^{JEPU}$			1.0000**(0.4534)	1.1766**(0.5001)	11.8825**(4.2401)

For the Chinese stock market, in models GARCH-MIDAS-RV, GARCH-MIDAS-RV-X and AO-GARCH-MIDAS-RV-X, all of them show a positive correlation between RV and volatility, but in model G-M-RV this relationship is very weak, while in the other two models it is significant at 1%. Similarly, CM2 also exhibits a significant positive effect at 5%, which be supported in GARCH-MIDAS-RV-X and AO-GARCH-MIDAS-RV-X models (not statistically significant in GARCH-MIDAS-X). Conversely, CER and CEPU exhibit significant negative impacts across all three models, indicating that China’s exchange rate and economic policy uncertainty substantially dampen long-run stock price volatility. Similarly, JRV also displays a significantly positive correlation with Japanese stock market volatility across three models. Although JM2 negative in GARCH-MIDAS and GARCH-MIDA-RV models, lacks statistical significance. While in AO-GARCH-MIDAS, JM2 is significantly positive at the 10% level. Conspicuously, JER and JEPU consistently exhibit a significant positive impact on Japanese stock volatility across all models, diverging from the findings in the Chinese stock market.

Overall, most parameter estimates for the long-run component *τ*_*t*_ demonstrate significance, indicating the enduring impact of money supply, exchange rates, and economic policy uncertainty on stock markets in both China and Japan. Notably, the AO-GARCH-MIDAS model outperforms other models, with a majority of parameter results exhibiting higher significance and significance levels.

To assess whether model performance can be enhanced through outlier correction, we scrutinize the estimation outcomes for the four aforementioned models across the full sample period. The evaluation of model fitting efficacy hinges on a direct comparison between observed and predicted daily volatility. Thus, this study employs four distinct loss functions: Mean Absolute Error (MAE), Mean Squared Error (MSE), Mean Absolute Deviation (MAD), and Mean Squared Deviation (MSD), which are denoted as follows

$$MAE=\frac{1}{n}\sum_{t=1}^{n}\left|\sigma_{t}-{\hat{\sigma }}_{t}\right|,$$

$$MSE=\frac{1}{n}\sum_{t=1}^{n}{\left(\sigma_{t}-{\hat{\sigma }}_{t}\right)}^{2},$$

$$MAD=\frac{1}{n}\sum_{t=1}^{n}\left|\sqrt{\sigma_{t}}-\sqrt{{\hat{\sigma }}_{t}}\right|,$$

$$MSD=\frac{1}{n}\sum_{t=1}^{n}{\left(\sqrt{\sigma_{t}}-\sqrt{{\hat{\sigma }}_{t}}\right)}^{2}.$$

Where n is the total number of volatility forecasts, *σ*_*t*_ and ${\hat{\sigma }}_{t}$ represent the actual value and forecast value of the volatility, respectively.

Table 3 presents the outcomes from the assessment of diverse model specifications, demonstrating consistent findings for both Japan and China. Primarily, the results obtained through the loss function analysis reveal that models incorporating low-frequency economic variables, namely GARCH-MIDAS-X, GARCH-MIDAS-RV-X, and AO-GARCH-MIDAS-RV-X, consistently exhibit diminished values in comparison to GARCH-MIDAS-RV and the standard GARCH model. This substantiates the assertion that integrating low-frequency economic variables significantly amplifies the model’s efficacy. Remarkably, the GARCH model consistently ranks the lowest across all evaluation criteria, with a substantial margin, notably in terms of Akaike Information Criterion (AIC) and Bayesian Information Criterion (BIC). Specifically, in Table 3, Panel A, its AIC and BIC values are more than twice those of the top-performing model. In Panel B, the gap between the GARCH model and the leading model is striking, with AIC and BIC disparities of 2484.345 and 2438.285, respectively. Moreover, the incorporation of realized volatility does not substantially influence the estimation process. Although GARCH-MIDAS-RV-X exhibits smaller values in the loss function, its AIC and BIC values are comparatively larger. Lastly, the AO-GARCH-MIDAS-RV-X model consistently outperforms the other four models, demonstrating superior performance with significantly lower loss function values and markedly smaller AIC and BIC scores. In sum, in the in-sample result, AO-GARCH-MIDAS-RV-X emerges as the most robust model, reflecting its superior predictive capacity and stability.

**Table 3:** GARCH-type full sample parameter estimation results.

Panel A In-sample results of Chinese loss functions and information standards						
Model	MAE	MSE	MAD	MSD	AIC	BIC
GARCH	0.3655	**0.7416**	0.3223	0.1768	4743.281	4767.661
GARCH-MIDAS-RV	0.3635	0.8548	0.3126	0.1758	3523.49	3558.553
GARCH-MIDAS-X	0.3588	0.8491	0.3099	0.1724	3495.888	3554.326
GARCH-MIDAS-RV-X	0.3572	0.7964	0.3132	0.1721	3855.604	3926.818
AO-GARCH-MIDAS-RV-X	**0.3081**	0.9406	**0.2646**	**0.1565**	**2063.513**	**2133.639**
Panel B In-sample results of Japanese loss functions and information standards						
Model	MAE	MSE	MAD	MSD	AIC	BIC
GARCH	0.3565	0.6748	0.3217	0.1655	5129.268	5153.372
GARCH-MIDAS-RV	0.3594	0.5966	0.3234	0.1668	3945.111	3980.193
GARCH-MIDAS-X	0.3590	0.5942	0.3230	0.1660	3940.422	3998.892
GARCH-MIDAS-RV-X	0.3392	**0.5318**	0.3134	0.1560	4461.464	4533.265
AO-GARCH-MIDAS-RV-X	**0.3093**	0.6526	**0.2793**	**0.1514**	**2644.923**	**2715.087**

### 4.2 Out-of-sample forecast evaluation of GARCH-type models

For market participants, the primary concern lies in the model’s capacity to predict future stock volatility rather than just in-sample performance [30,38,39]. Because of the prevalent desire among investors to seek new investment insights from historical market data, there is a pressing need for models characterized by improved efficiency to facilitate their effective exploration of this valuable information.

In light of this objective, this section delves into an analysis of whether the incorporation of a model with additive outliers can augment their predictive prowess. To ensure the robustness of our findings and mitigate the influence of extraneous variables on forecasting results, we employ a unified forecasting method, characterized by a rolling window approach consisting of five steps. This approach encompasses parameter estimation for GARCH-type models, involving the analysis of model parameters utilizing sample data spanning various periods, followed by out-of-sample forecasting. Evidently, the overall estimation timeline is iteratively adjusted for each forecasting outcome generated within the confines of this recursive framework. In particular, we commence by partitioning the complete dataset for both China and Japan into two separate subgroups. The complete dataset sourced from the two nations was initially partitioned into distinct subgroups. The complete dataset from both countries is initially segregated into two distinct subgroups. The allocation comprises 80% designated for in-sample analysis and 20% earmarked for out-of-sample examination, with a total of 660 forecast periods. In the case of China, the in-sample estimation spans from January 18, 2013, to August 5, 2020, while the out-of-sample forecast period extends from August 6, 2020, to March 31, 2023. Similarly, for Japan, the in-sample estimation period encompasses data from December 26, 2012, to July 13, 2020, followed by the corresponding out-of-sample evaluation period spanning from July 14, 2020, to March 31, 2023.

The evaluation of out-of-sample model forecasting performance also relies on four distinct loss functions; however, the predictive efficacy of the model displays noteworthy disparities between China and Japan. As delineated in Examining the loss functions associated with the GARCH-MIDAS-RV and GARCH-MIDAS-X models in Table 4, Panel B reveals that the model incorporating low-frequency macro factors and EPU encompasses more valuable predictive information. Notably, the AO-GARCH-MIDAS-RV-X model, focusing on additive outliers, notably surpasses the other models in terms of predictive accuracy. Specifically, across both Chinese and Japanese datasets, the MAE, MSE, MAD, and MSD values are minimized. In summary, this innovative model exhibits unequivocal superiority, excelling in both in-sample estimation and out-of-sample prediction.

**Table 4:** Out-of-sample prediction assessment results.

Panel A Out-of-sample forecast validation of GARCH–type models, China						
Model	MAE	MSE	MAD	MSD	Mean Rank	Theil’s U
GARCH	0.6900	1.3163	0.3265	0.1871	0.6300	0.2537
GARCH-MIDAS-RV	0.7072	1.300	0.3398	0.1941	0.6353	0.2487
GARCH-MIDAS-X	0.7372	1.3513	0.3535	0.2046	0.6617	0.2570
GARCH-MIDAS-RV-X	0.7056	1.3538	0.3426	0.2039	0.6515	0.2573
AO-GARCH-MIDAS-RV-X	**0.6036**	**1.2259**	**0.2948**	**0.1614**	**0.5714**	**0.2448**
Panel B Out-of-sample forecast validation of GARCH–type models, Japan						
Model	MAE	MSE	MAD	MSD	Mean Rank	Theil’s U
GARCH	0.8080	1.0915	0.3692	0.2066	0.6188	0.2391
GARCH-MIDAS-RV	0.8046	1.0839	0.3679	0.2053	0.6154	0.2383
GARCH-MIDAS-X	0.7680	1.0728	0.3493	0.1915	0.5954	0.2371
GARCH-MIDAS-RV-X	0.8559	1.2118	0.3844	0.2234	0.6689	0.2520
AO-GARCH-MIDAS-RV-X	**0.7390**	**1.0390**	**0.3426**	**0.1904**	**0.5778**	**0.2333**

Table 4, Panel A, the GARCH model exhibits smaller values across all four loss functions compared to the GARCH-MIDAS-X and GARCH-MIDAS-RV-X models, which show robust out-of-sample forecasting capabilities, despite their underperformance in the in-sample context. Remarkably, in Japan, the GARCH model does not establish an unequivocal advantage. Moreover, the performance of GARCH-MIDAS-X and GARCH-MIDAS-RV-X models diverges significantly between the two countries, with the latter model slightly outperforming predictions in China but lagging behind all models in Japan. This disparity implies that the incorporation or exclusion of the low-frequency factor RV does not singularly enhance the model’s predictive prowess. Examining the loss functions associated with the GARCH-MIDAS-RV and GARCH-MIDAS-X models in Table 4, Panel B reveals that the model incorporating low-frequency macro factors and EPU encompasses more valuable predictive information. In addition, the tables presented offer consistent findings, affirming the exceptional predictive efficacy of the AO-GARCH-MIDAS-X model. This is evidenced not only through individual loss functions but also through the aggregate of four distinct loss functions. The model’s unparalleled performance is distinctly evident across both in-sample estimation and out-of-sample prediction, underscoring its unequivocal superiority in predictive accuracy and innovation.

In addition to using these four loss functions to assess the predictive effectiveness of the model, this study uses Theil’s U statistic. This statistical metric scales the Root Mean Square Error (RMSE) by accounting for the variability inherent in the underlying data, thus offering the advantage of independence from the actual process variance. The formula for Theil’s U statistic is as follows:

$$U=\frac{\sqrt{{\left(T\right)}^{-1}\sum_{i}^{T}{\left(y_{i}^{a}-y_{i}^{f}(n)\right)}^{2}}}{\sqrt{{\left(T\right)}^{-1}\sum_{i}^{T}{\left(y_{i}^{a}-y_{i}^{*}(n)\right)}^{2}}}$$

Where, T represents the quantity of forecasting periods under scrutiny. $y_{i}^{a}$ signifies the authentic value while $y_{i}^{*}$ indicates the anticipated value derived from the naïve forecast for 5 steps into the future, with this study employing the no-change forecast as the basis for the naïve forecast. $y_{i}^{f}$ is the ith projected output for five steps in the future. When the value of Theil’s U exceeds 1, it indicates a scenario wherein the forecast model’s performance worse compared to the naïve forecast.

The final column of Table 4 presents the Theil’s U values. It is noteworthy that all values are substantially below 1, indicating that the predictive capabilities of the models chosen for this study surpass those of the naïve forecast. Furthermore, the Theil’s U value associated with the AO-GARCH-MIDAS-RV-X model emerges as the smallest in both countries, a result consistent with findings observed across the other four loss functions.

Table 5 provides a comprehensive overview of model performance, encompassing evaluation across four distinct error measures. The initial four columns showcase the mean ratings extracted from Table 4, while the last column aggregates the average mean ratings for each method. Analysis of this table distinctly illustrates the sustained competitive edge of the GARCH-MIDAS model, particularly with outlier correction, across all four assessment criteria. Notably, in the overall rankings, this model confidently secures the top position, closely pursued by the GARCH model.

**Table 5:** Summary of the ranking of methods in Table 4 for the two stock indices.

Model	Mean Rank for MAE	Mean Rank for MSE	Mean Rank for MAD	Mean Rank for MSD	Mean of Mean Ranks
GARCH	0.7490	1.2039	0.3479	0.1969	0.6244
GARCH-MIDAS-RV	0.7559	1.1920	0.3539	0.1997	0.6254
GARCH-MIDAS-X	0.7526	1.2121	0.3514	0.1981	0.6286
GARCH-MIDAS-RV-X	0.7808	1.2828	0.3635	0.2137	0.6602
AO-GARCH-MIDAS-RV-X	**0.6713**	**1.1325**	**0.3187**	**0.1759**	**0.5746**

This study employs four distinct loss functions to gauge the predictive efficacy of GARCH-type models in volatility forecasting. The utilization of multiple criteria enhances the efficiency of our analysis [40]. Moreover, to systematically appraise the predictive performance of these models based on loss functions, the study employs the model confidence set (MCS) test [41]. This test offers a straightforward and rapid method for comparison, bypassing the necessity to establish a baseline model when assessing the predictive accuracies of diverse models.

Tables 6 and 7 present the selected optimal superior models (SSM), determined through assessments of absolute and squared prediction errors, respectively. Notably, the associated p-values demonstrate significance at the 20% level. Within these findings, the AO-GARCH-MIDAS-RV-X model emerges as the least eliminated, closely followed by the GARCH-MIDAS-RV-X model. This observation accentuates the diminishing predictive efficacy of models in the presence of outliers. Consequently, it underscores the imperative for heightened consideration of outlier influence when employing models for predictive purposes.

**Table 6:** Composition of remaining models in the superior set for two stock indices using absolute forecast errors.

	China		Japan	
Model	Tmax,M	Rank_M	Tmax,M	Rank_M
GARCH				
GARCH-MIDAS-RV			0.7004	3
GARCH-MIDAS-X				
GARCH-MIDAS-RV-X	1.0000	1	0.9774	2
AO-GARCH-MIDAS-RV-X	0.9394	2	1.0000	1

**Table 7:** Composition of remaining models in the superior set for two stock indices using squared forecast errors.

	China		Japan	
Model	Tmax,M	Rank_M	Tmax,M	Rank_M
GARCH	0.9994	3	0.9294	4
GARCH-MIDAS-RV	0.8060	4	1.0000	2
GARCH-MIDAS-X	0.5384	5		
GARCH-MIDAS-RV-X	1.0000	2	0.9658	3
AO-GARCH-MIDAS-RV-X	1.0000	1	1.0000	1

From the results of the MCS test, the predictive ability of the GARCH-MIDAS class of models generally outperforms the standard GARCH model, this finding is similar to Liu, Zhang [42]. They employ both GARCH-MIDAS model and a variety of GARCH models for predicting EUA futures, concluding that the former has stronger predictive power. In addition, GARCH-MIDAS model that includes RV and low-frequency macroeconomic variables can improve its predictive capability. The findings of Asgharian, Hou [12] support this conclusion. Finally, this study finds that the inclusion of outlier correction function in the model results in a significant improvement in predictive ability. This finding is consistent with Chen and Liu [34] and Franses and Ghijsels [7]. After adding this function to the ARMA and GARCH models, Chen and Li find that the predictive effect of the model on the volatility is significantly better than its standard model.

**Fig 2:**
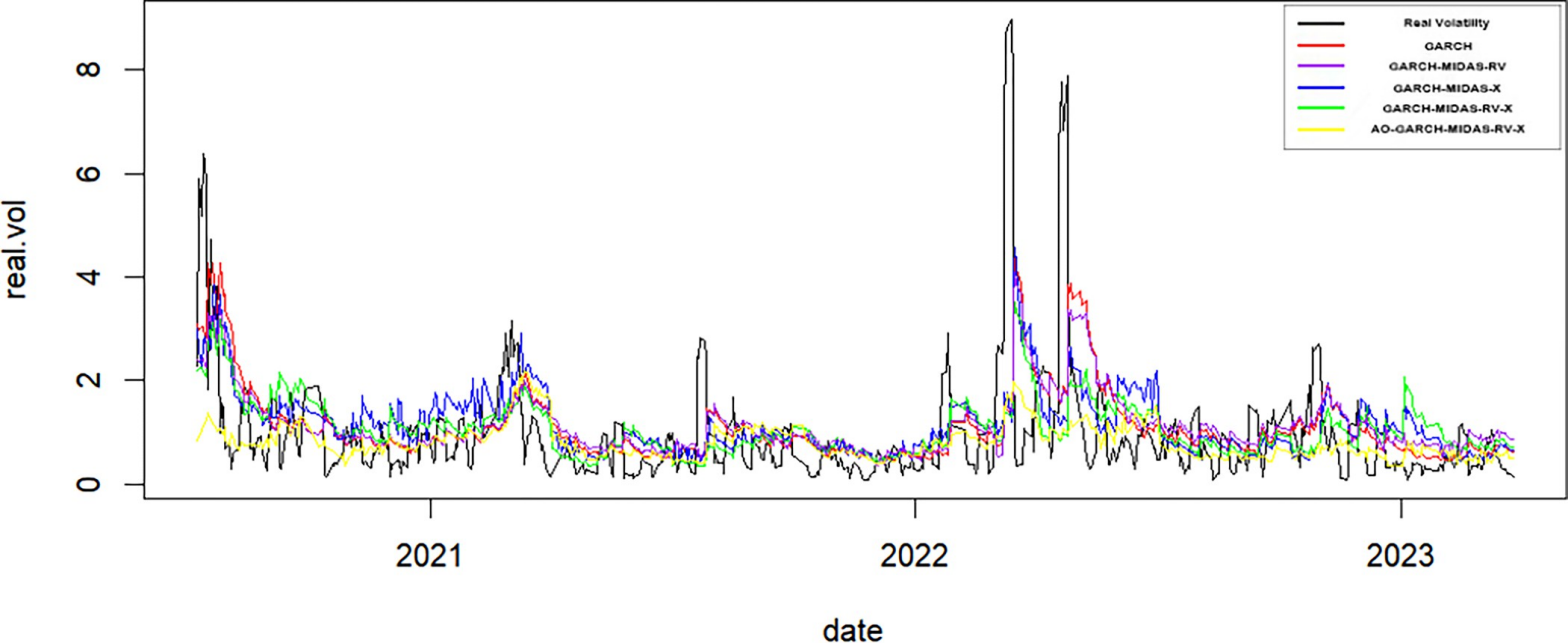
The forecasting secular volatilities of GARCH-type models, China.

**Fig 3:**
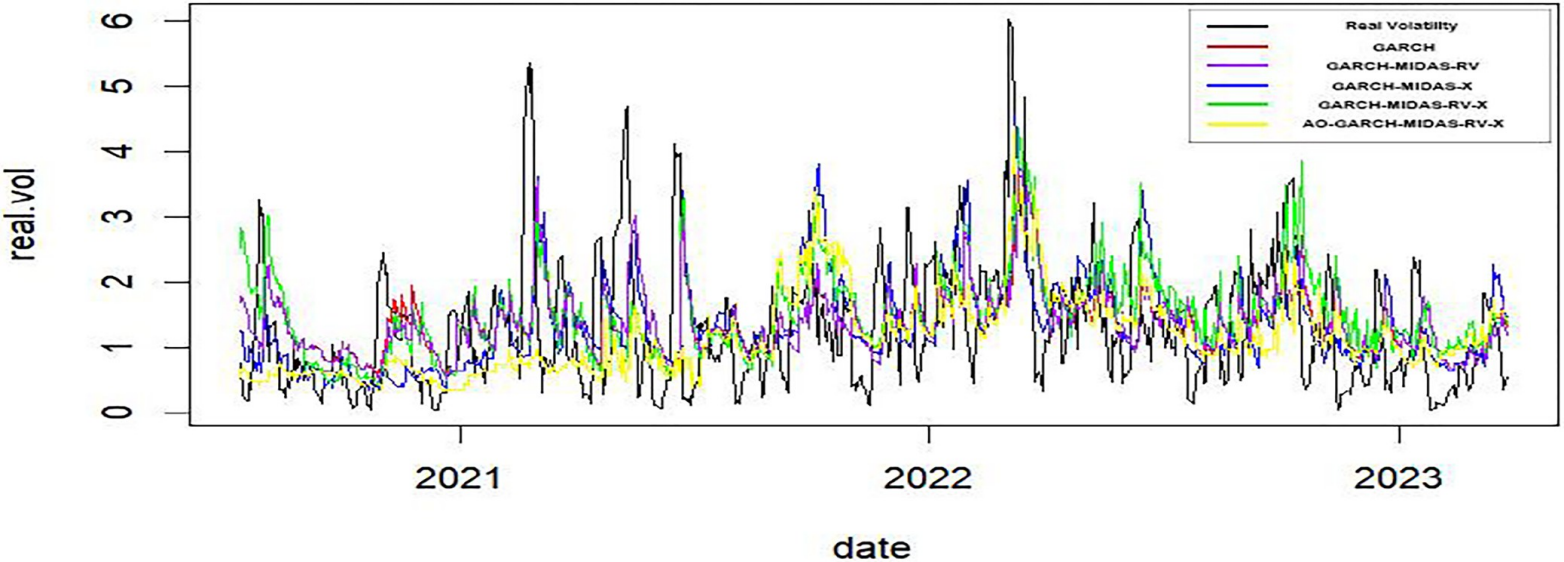
The forecasting secular volatilities of GARCH-type models, Japan.

As delineated in Figs 2 and 3, it becomes apparent that the predictive outcomes of the AO-GARCH-MIDAS-RV-X model exhibit greater proximity to actual volatility levels. Furthermore, the observation that the temporal lag in predictions is notably diminished during periods marked by extreme volatility.

In pursuit of bolstering the robustness of our conclusions, we have undertaken an additional assessment utilizing the Diebold and Mariano (DM) test [43] to gauge the comparative predictive accuracy of the various models. Then, we define the mean loss differential of the time series as a critical metric in our analysis.


$$\bar{d}=\frac{1}{T}\sum_{t=1}^{T}d_{t}=\frac{1}{T}\sum_{t=1}^{T}\left[{L(e}_{i,t})-{L(e}_{j,t})\right]$$

Where *L*(*e*_*i*,*t*_) and *L*(*e*_*j*,*t*_) are respectively the absolute error loss from two different competing models.

The Diebold-Mariano test can be expressed as:

$$DM=\frac{\bar{d}}{\sqrt{\frac{1}{T}Var(d)}}∼N(0,1)$$

Note that the *Var*(*d*) is a consistent estimator of the asymptotic variance of *d*_*t*_. The null hypothesis posits an equivalence in predictive accuracy between the alternative model and the benchmark model, while the alternative hypothesis postulates a better predictive accuracy for the benchmark model. In our analysis, we employ AO-GARCH-MIDAS-RV-X as the reference benchmark, subjecting each alternative model to a sequential comparative evaluation. The outcomes of these comparisons are presented in Table 8 for reference.

**Table 8:** Results of the diebold–Mariano test.

Model	China	Japan
GARCH	3.9264*** (0.0000)	3.592*** (0.0002)
GARCH-MIDAS-RV	5.1586*** (0.0000)	3.4617*** (0.0003)
GARCH-MIDAS-X	7.0646*** (0.0000)	1.5815* (0.0571)
GARCH-MIDAS-RV-X	5.9332*** (0.0000)	5.9405*** (0.0000)

Note: The table presents the evaluation results between the AO-GARCH-MIDAS-RV-X and various other GARCH-type models. An indication of a ratio exceeding 1 signifies that the predictive accuracy of the model under consideration is comparatively inferior to that of the benchmark model. The accompanying Diebold and Mariano (DM) test p-values are provided within parentheses, with asterisks serving as indicators of statistical significance levels. Specifically

* denotes significance at the 10% level, and

** signifies significance at the 1% level.

### 4.3 Robustness analysis

Numerous studies have confirmed that the selected length of the prediction window leads to differences in experimental results [44–46]. As previous predictions covered the pandemic period, in order to capture more clearly whether the predictive ability of the model is robust or not, this section will separately explore the sample data without the COVID-19 component. Based on previous studies that typically used January 2020 as the pandemic start date [47], this study divided the data prior to the onset of the pandemic into the period from October 2009 to December 2019, with a prediction period of 500 days

Table 9 demonstrates the prediction performance of each model in China and Japan before the outbreak. In China, the predictive ability of each model is not unanimously confirmed, among which the predictive ability of AO-GARCH-MIDAS-RV-X is only supported by MAE and MAD. However, according to the results of the DM test in Table 10, the p-values are all found to be less than 10% when using this model as the baseline model in comparison with the other competing models, indicating that the predictive ability of this model is significantly better than that of all the competing models. The findings depicted in Tables 9 and 10 regarding the different models in predicting Japanese stock volatility are in strong agreement. Firstly, the AO-GARCH-MIDAS-RV-X model corresponds to the smallest values of the four loss functions and Theil’s U, closely followed by the GARCH-MIDAS-RV-X model. Secondly, all p-values in the DM test are significant at the 1% level, indicating that the AO-GARCH-MIDAS-RV-X has the strongest predictive power. Overall, the results of the robustness test are consistent with the above findings that this model has a stable out-of-sample predictive ability. Table 10 summarizes the means of the loss functions from the two stocks. For the AO-GARCH-MIDAS-RV-X model, the means of the three loss functions are significantly smaller than the other models, except for MSE. The mean values of the four loss functions corresponding to each model are shown in the last column of the table, again demonstrating that the innovative models excel in forecasting. Table 11 shows the results of DM-test. All the tests are significant, which means that all the models fail to beat the AO-GARCH-MIDAS-RV-X model in terms of forecasting performance, both in the Chinese and Japanese stock markets.

**Table 9:** Out-of-sample forecasting results in pre COVID-19 period.

Panel A Prediction results in pre COVID-19 period, China					
Model	MAE	MSE	MAD	MSD	Theil’s U
GARCH	0.8933	**2.0953**	0.3675	0.2487	**0.2868**
GARCH-MIDAS-RV	1.0004	2.1763	0.4123	0.2750	0.2923
GARCH-MIDAS-X	0.8759	2.1157	0.3638	**0.2468**	0.2882
GARCH-MIDAS-RV-X	0.8962	2.2584	0.3762	0.2699	0.2978
AO-GARCH-MIDAS-RV-X	**0.8377**	2.4709	**0.358**	0.2856	0.3115
Panel B Prediction results in pre COVID-19 period, Japan					
Model	MAE	MSE	MAD	MSD	Theil’s U
GARCH	0.9529	1.9489	0.4444	0.2973	0.3286
GARCH-MIDAS-RV	0.9462	1.9242	0.4493	0.3102	0.3265
GARCH-MIDAS-X	0.9921	1.9322	0.4665	0.3212	0.3272
GARCH-MIDAS-RV-X	0.9106	1.7674	0.4358	0.2904	0.3129
AO-GARCH-MIDAS-RV-X	**0.7686**	**1.6169**	**0.3775**	**0.2438**	**0.2993**

**Table 10:** Summary of the ranking of methods in Table 9 for the two stock indices.

Model	Mean Rank for MAE	Mean Rank for MSE	Mean Rank for MAD	Mean Rank for MSD	Mean of Mean Ranks
GARCH	0.9231	2.0221	0.4060	0.2730	0.9060
GARCH-MIDAS-RV	0.9733	2.0503	0.4308	0.2926	0.9367
GARCH-MIDAS-X	0.9340	2.0240	0.4152	0.2840	0.9143
GARCH-MIDAS-RV-X	0.9034	**2.0129**	0.4060	0.2802	0.9006
AO-GARCH-MIDAS-RV-X	**0.8032**	2.0439	**0.3681**	**0.2647**	**0.8700**

**Table 11:** Results of the diebold–Mariano test in pre COVID-19 period.

Model	China	Japan
GARCH	1.7845**(0.0375)	6.5827***(0.0000)
GARCH-MIDAS-RV	4.5005***(0.0000)	6.6514***(0.0000)
GARCH-MIDAS-X	1.4384*(0.0755)	8.0824*(0.0000)
GARCH-MIDAS-RV-X	2.3646***(0.0092)	5.7967*(0.0000)

Note: The table presents the evaluation results between the AO-GARCH-MIDAS-RV-X and various other GARCH-type models. An indication of a ratio exceeding 1 signifies that the predictive accuracy of the model under consideration is comparatively inferior to that of the benchmark model. The accompanying DM test p-values are provided within parentheses, with asterisks serving as indicators of statistical significance levels. Specifically

* denotes significance at the 10% level, and

*** signifies significance at the 1% level.

## 5. Conclusions

This study delves into the substantial influence of money supply, exchange rates, and economic policy uncertainty on stock market volatility prediction through the GARCH-type model. The findings furnish noteworthy insights applicable to both the Chinese and Japanese stock markets, albeit with discernible performance disparities between the two nations. Among the variables, only M2 consistently demonstrates a contributory effect on stock volatility in both countries. Conversely, the impact of ER and EPU diverges significantly: in China, these variables exhibit a substantial negative influence on future volatility, whereas in Japan, they showcase a positive effect. Furthermore, employing four distinct loss functions (MAE, MSE, MAD, and MSD), we juxtapose the model’s estimation prowess pre and post the inclusion of macroeconomic variables. Results underscore a noteworthy enhancement in the model’s estimation capability post incorporation of these variables, underscoring the utility of low-frequency economic factors in estimating stock volatility. Subsequently, the in-sample explanatory strength of the AO-GARCH-MIDAS model, equipped with corrected outliers, remains robust.

However, out-of-sample empirical results reveal nuances: GARCH-MIDAS-RV-X, incorporating realized volatility, exhibits superior predictive power over GARCH-MIDAS-X in China, while the GARCH-MIDAS-X model, integrating macroeconomic variables and EPU, outperforms the GARCH-MIDAS-RV model in predicting Japanese stock volatility. Comparative assessments against rival models reaffirm the superior forecasting accuracy of the AO-GARCH-MIDAS model. The consistency between the MCS test and DM test bolsters the robustness of our main findings.

In summary, this research introduces novel approaches to forecast stock volatility, enriching the landscape of forecasting methodologies. The implications of our findings extend to policymakers and stock market participants. Policymakers should consider potential market alterations resulting from relevant policies, emphasizing the need to maintain market stability and transparency. Simultaneously, market participants can leverage these insights to mitigate risks and make informed investment decisions.
